# Glycemic Analysis and Stratification of Pediatric Patients with Type 1 Diabetes Using isCGM in Southern Spain: Insights from the Andiacare Digital Platform

**DOI:** 10.3390/jcm14176243

**Published:** 2025-09-04

**Authors:** Isabel Leiva-Gea, Fernando Moreno-Jabato, Ana Belén Ariza-Jiménez, Alfonso Lendínez-Jurado, Ana Gómez-Perea, María del Mar Romero-Pérez, Emilio García-García, María Ángeles Santos Mata, Gabriela Martínez-Moya, Jerónimo Momblan, Alfonso María Lechuga-Sancho, José María Gómez-Vida, Mercedes Mier-Palacios, María del Pilar Ranchal-Pérez, Gustavo Vivas-González, Patricia Calleja Cabeza, Eugenio Fernández-Hernández, Ana Pilar Jiménez-Martín, Jessica Guarino-Narváez, Pablo Rodríguez de Vera-Gómez, María Asunción Martínez-Brocca

**Affiliations:** 1 Department of Pediatric Endocrinology, Regional University Hospital of Málaga, 29011 Málaga, Spain; isabeleivag@gmail.com (I.L.-G.); gomezpereana@gmail.com (A.G.-P.); 2 Instituto de Investigación Biomédica de Málaga (IBIMA)-Plataforma BIONAND, 29010 Málaga, Spain; jabato@pacalcs.com; 3 Department of Biomedicine and Dentistry, Faculty of Biomedical Sciences and Sports, Universidad Europea de Andalucía, 29010 Málaga, Spain; 4 PACAL Care Systems, 29006 Málaga, Spain; 5 Pediatric Endocrinology Service, Reina Sofía University Hospital, Avda. Menendez Pidal, s/n, 14004 Córdoba, Spain; anab.ariza.sspa@juntadeandalucia.es; 6 Growth Study Group, Pediatric Endocrinology and Nutrition, Maimonides Institute of Biomedical Research of Cordoba, Av. Menendez Pidal, s/n, 14004 Córdoba, Spain; 7 Faculty of Medicine and Nursing, University of Cordoba, Avda. Menendez Pidal, 7, 14004 Córdoba, Spain; 8 Distrito Sanitario Málaga-Guadalhorce, 29009 Málaga, Spain; 9 Department of Pediatric Endocrinology, Virgen Macarena University Hospital, 41009 Sevilla, Spain; mmromeroperez@hotmail.com; 10 Department of Pediatric Endocrinology, Virgen del Rocío University Hospital, 41013 Sevilla, Spain; ejgg67@gmail.com; 11 Department of Pediatric Endocrinology, University Hospital of Jerez de la Frontera, 11407 Cádiz, Spain; mangeles.santos.sspa@juntadeandalucia.es; 12 Department of Pediatric Endocrinology, University Hospital of Jaén, 23007 Jaén, Spain; gabriela.martinez.sspa@juntadeandalucia.es; 13 Department of Pediatric Endocrinology, Torrecárdenas Hospital, 04009 Almería, Spain; jeronimo.momblan.sspa@juntadeandalucia.es; 14 Department of Pediatric Endocrinology, Puerta del Mar University Hospital, 11009 Cádiz, Spain; alfonso.lechuga@gm.uca.es; 15 Department of Maternal and Child Health and Radiology, School of Medicine, University of Cádiz, 11003 Cádiz, Spain; 16 Biomedical Research and Innovation Institute of Cádiz (INiBICA), Puerta del Mar University Hospital, 11009 Cádiz, Spain; 17 Department of Pediatric Endocrinology, San Cecilio Clinical University Hospital, 18016 Granada, Spain; gomezvida@gmail.com; 18 Department of Pediatric Endocrinology, Juan Ramón Jiménez University Hospital, 21005 Huelva, Spain; mmmier_7@hotmail.com; 19 Department of Pediatric Endocrinology, Costa del Sol Hospital, 29603 Marbella, Spain; mariapilar.ranchal.p.sspa@juntadeandalucia.es; 20 Department of Pediatric Endocrinology, Serranía de Ronda Hospital, 29400 Ronda, Spain; gustavovivasg@gmail.com; 21 Department of Pediatric Endocrinology, Alto Guadalquivir Hospital, 23740 Andújar, Spain; patcalleja@yahoo.es; 22 Department of Pediatric Endocrinology, Instituto Hispalense de Pediatría, 41013 Sevilla, Spain; euferer@ihppediatria.com; 23 Department of Pediatric Endocrinology, Infanta Margarita Hospital, 14940 Cabra, Spain; apjimenezmartin@hotmail.com; 24 Department of Pediatric Endocrinology, Punta de Europa Hospital, 11207 Algeciras, Spain; jessica.guarino.sspa@juntadeandalucia.es; 25 Department of Endocrinology and Nutrition, Virgen Macarena University Hospital, 41009 Seville, Spain; pablo.rodriguezvera.sspa@juntadeandalucia.es (P.R.d.V.-G.); masuncion.martinez.sspa@juntadeandalucia.es (M.A.M.-B.)

**Keywords:** type 1 diabetes mellitus, pediatric population, advanced technologies and treatments for diabetes (AATD), continuous glucose monitoring (CGM), multiple daily injections of insulin (MDI), therapeutic scalability, time in range (TIR)

## Abstract

**Background/Objectives**: Type 1 diabetes mellitus (T1D) is the most common metabolic disorder in children, with significant physical and emotional impacts. Achieving optimal glucometric control is challenging due to the complex management and limitations of insulin therapy. Advances in pharmacology and technology, including continuous glucose monitoring (CGM) systems, offer new options for diabetes management. We developed Andiacare, an open-source platform for macro/micro-management of diabetes and analyzed its application in a pediatric T1D cohort to evaluate glucometric control patterns. **Methods**: A retrospective cohort study was conducted in a pediatric population (<18 years old) in Andalusia, Spain. Patients treated with Multiple Daily Injections of Insulin (MDI) and FreeStyle Libre 2 System (Abbott, Spain) were included. The patient data were analyzed using the Andiacare platform, which categorizes patients based on the Advanced Technologies and Treatments for Diabetes (ATTD) panel’s targets for glucometric control. **Results**: The study included 2215 patients from 18 pediatric hospitals. The Andiacare platform categorized patients into four groups based on glucometric control parameters, enabling patient stratification based on their glucometric control. Only 25.8% of the cohort achieved the recommended Time in Range (TIR), and 9.5% of the patients achieved all target parameters of glucometric control. Age is a determinant factor in adherence and achievement of set goals. **Conclusions**: This study offers insights into glucometric control in a large pediatric population with T1D in Andalusia. Few patients achieved the recommended glucometric control targets, highlighting the need for improved management strategies. The use of digital platforms such as Andiacare might contribute to facilitating the management of large pediatric cohorts. New algorithms integrating glucometric and non-glucometric parameters are required for improved individual and cohort categorization to optimize therapeutic interventions.

## 1. Introduction

The most common metabolic disorder in children is type 1 diabetes mellitus (T1D), having a great physical and emotional toll [1]. Most of the patients show a suboptimal glucometric control that can be attributed to the complexity of the management and the limitations of insulin therapy’s ability to replicate beta cell function which causes stress for patients and caregivers. In addition, there are factors involved that can be improved, such as knowledge of the disease, physical activity, diet, and self-care [2].

Patients are encouraged to monitor glucose levels and maintain blood glucose levels within the normal range through constant and frequently complex decisions on insulin treatment based on dietary measures, physical activity, and glucose levels in order to prevent acute and chronic complications [2]. The Diabetes Control and Complications Trial established the importance of attaining optimal glucometric control in participants with T1D to delay and potentially avoid long-term diabetes complications [3,4].

Recent advancements in pharmacology, including the development of newer insulin analogs, coupled with technological innovations, have substantially expanded the repertoire of options and flexibility available for diabetes management. Breakthroughs in technology, such as interstitial glucose monitoring through real-time continuous glucose monitoring (rtCGM), intermittently scanned continuous glucose monitoring (isCGM), pump therapy, sensor-augmented pump therapy, and hybrid closed-loop systems, have revolutionized diabetes care [4,5].

Studies conducted throughout SWEET centers (multinational network of centers providing care for children, adolescents, and young adults with diabetes), have also confirmed the increased use of pumps, rtCGM and isCGM among pediatric and young adult patients [4]. The average number of patients with T1D who have used CGM increased from 7% in 2010–2012 to 30% in 2016–2018 and 43.2% in 2021 [6,7].

Recent upswings in the use of interstitial glucose monitoring technologies have given people with diabetes and healthcare professionals unprecedented access to a range of new indicators of glucose control. Some of these metrics are useful research tools and others have been welcomed by patient groups for providing insights into the quality of glucose control not captured by conventional laboratory testing [8]. There are four metrics from CGM that are of clinical value for people with diabetes and healthcare professionals as they are reflective of diabetes management in clinical practice: Time In Range (TIR), Time Below Range (TBR), Time Above Range (TAR), and coefficient of variation in glucose (CV). TIR has emerged as a clinically meaningful glycemic target, and achieving TIR > 70% is currently endorsed by international consensus guidelines, including the Advanced Technologies & Treatments for Diabetes (ATTD). TIR is also increasingly recognized as a surrogate endpoint for HbA1c, providing more dynamic and individualized information on glycemic control [8,9,10].

In addition to its individual applications, CGM represents a highly valuable tool for assessing glucometric control in individuals with type 1 diabetes, both at an individual level and within broader diabetes populations or cohorts. Several tools have been proposed to aid in this analysis, with the Glycemic Risk Index (GRI) being particularly notable. The GRI is a metric designed to assess glucose quality in diabetes subjects based on CGM data. It is based on the combination of two essential components: hypoglycemia and hyperglycemia, weighted according to their clinical impact. The variables used include the following:

(I) Time in very low hypoglycemia (<54 mg/dL);
(II) Time in low hypoglycemia (54–70 mg/dL);
(III) Time in high hyperglycemia (180–250 mg/dL) and;
(IV) Time in very high hyperglycemia (>250 mg/dL).

The algorithm combines weighted values of time in each range, adjusting the weights based on manual classification of individuals made by specialists. Accordingly, the algorithm assigns greater weight to extreme values of hypoglycemia and hyperglycemia, considering the clinical relevance of these fluctuations. It is computed as GRI = (3.0 × %TBR < 54) + (2.4 × %TBR 54–70) + (1.6 × %TAR > 250) + (0.8 × %TAR 180–250), where the maximum possible score is capped at 100. The GRI is designed to estimate clinicians’ percentile rankings, with 0 representing optimal control and 100 indicating the poorest control [11]. For clinical visualization, GRI values are stratified into five color-coded zones:

- Zone A (Green; 0–20);
- Zone B (Yellow; 21–40);
- Zone C (Orange; 41–60);
- Zone D (Light red; 61–80);
- Zone E (Dark red; 81–100).

Several studies demonstrate that the GRI is a useful metric for assessing the overall risk of hypoglycemia and hyperglycemia in people with T1D, across both pediatric and adult populations. This index provides a more comprehensive interpretation of metabolic control by integrating multiple glucometric variables into a single parameter [12,13,14].

The region of Andalusia, which covers an area of 87,597 km^2^ in the south of Spain, has a population of 8.4 million (18% of the country’s population). The Andalusian Public Health System (APHS) is responsible for the provision of universal healthcare in the region. This area has a known incidence of pediatric T1D of 20.8/105 h-y (0–4 years): 14.3/105 h-y; 5–9 years: 23.5/105 h-y; and 10–14 years: 25.2/105 h-y, respectively [15].

The aims of the study are to describe and analyze, at both individual and population levels, the key glycemic indicators (TIR, TAR, TBR) in a pediatric cohort with T1D using isCGM, based on anonymized data extracted from the Andiacare digital platform.

## 2. Materials and Methods

### 2.1. Study Design

A historical cohort population-based study was conducted within the pediatric population (under 18 years old) in the region of Andalusia.

### 2.2. Cohort Description

Patients with confirmed T1D were included in the study. Inclusion of patients from each center was conducted by the principal investigator. All pediatric patients included in the study were undergoing treatment with Multiple Daily Injections of Insulin (MDI) and isCGM with FreeStyle Libre 2 System (Abbott Diabetes Care 1360 South Loop Road Alameda, CA, USA). Patient data were extracted anonymously from the LibreView platform and included the analysis of the last 14 days at a single time point dated October 2022. A 14-day CGM data window was selected following the recommendations of the International Consensus on Time in Range, which indicate that this period, together with ≥70% sensor usage, is sufficient to reliably estimate long-term glycemic control [9]. All metrics (%TIR, %TBR, %TAR) were derived from absolute cumulative times (summed across the 14-day period) divided by the total monitored time. This method ensures consistency, as the exported CSV data from LibreView reports absolute values rather than daily averages.

### 2.3. Ethics

The study was conducted in accordance with the Declaration of Helsinki and the International Conference on Harmonization Good Clinical Practice Guidelines. Regarding the ethical approval for our study, due to the large-scale, anonymized nature of the dataset used, the Ethics Committee of the Regional Hospital of Malaga and the Regional Ethics Committee of Andalusia granted a waiver for individual informed consent in accordance with applicable regulations (Ethics Code: 0788-N-19, 30 July 2020).

### 2.4. Patient and Public Involvement

The design of the study was shared with patient associations and implemented incorporating feedback from patients and their families from the design. The findings of the study have been disseminated at patient forums at both local and national levels.

### 2.5. Andiacare Platform

The Andiacare platform has been designed for the categorization of patients included in the cohort. Andiacare Pathways are a set of algorithms that attempts to classify patients to lead strategic medical actions on an individual or a cohort. With the goal of giving patients with T1D intelligent agenda management capabilities, the first Andiacare algorithm was created. It contains several modules that form a main flow and two optional features. The main workflow starts with the following:

- File format detection and loading of structured patient data.
- Filtering patients by quality of their data, proceeding to eliminate those that contain erroneous entries or missing data in fields marked as essential. To ensure the quality of the analyzed data, automatic exclusion criteria were applied through the Andiacare platform. Patients with sensor usage time < 70% were excluded.
- ATTD19 Target Check: Following the ATTD19 targets for time in range, under range, or over range, a simple compliance or non-compliance check is performed for each patient.
- Calculation of statistics of the main fields, being able to segment sub-cohorts by age ranges, health centers of origin or objective criteria ATTD19.
- Patient classification: the system applies as many algorithms as requested.

For the categorization of the patients, the consensus on the cut-off points of adequate glucometric control for T1D pediatric patients of the Advanced Technologies and Treatments for Diabetes (ATTD) panel was used [4,9]. Glucometric targets for the pediatric population include seven key measurements of target glucose levels over a 24 h period: >70% of the time spent between 70 and 180 mg/dL of glycemia (Time In Range, TIR), <25% of the time spent between 180 mg/dL and 250 mg/dL (Time Above Range Level 1, TAR1), <5% of the time spent above 250 mg/dL (Time Above Range Level 2, TAR2), <4% of the time spent between 54 and 70 mg/dL (Time Below Range Level 1, TBR1), <1% of the time spent below 54 mg/dL (Time Below Range Level 2, TBR2) and <36% coefficient of variation (CV), as proposed by the Advanced Technologies and Treatments for Diabetes panel [9].

Andiacare algorithm classified the patients in four categories:

(I) Green: TIR > 70%, TBR1 < 4% and TAR1 < 25%.
(II) Yellow: 40% < TIR ≤ 70% or 4% < TBR1 < 11% or 25% ≤ TAR1 < 50%.
(III) Orange: 25% ≤ TIR < 40% or 11% ≤ TBR1 < 20% or 50% ≤ TAR1 < 75%.
(IV) Red: TIR < 25% or TBR1 > 20% or TAR1 ≥ 75%.

These thresholds were established through consensus panels involving diabetes teams from the participating hospitals (comprising both pediatric and adult patients). Notably, only the green category aligns with all three consensus cut-off points, while the other colors progressively deviate from it.

Defined analysis strategy using Andiacare was implemented into an R-package that included functionalities such as (I) loading and handling of cohort/individual data; (II) data checking and filtering for quality; (III) measurement of main variables statistics and ATTD targets accomplishments; (IV) comparison of classification algorithms (Andiacare and GRI); and (V) providing cohort reports. Code structure and strategy were designed to allow algorithms modification and/or inclusion by other contributors. Because of the specificity system source of our data (LibreView), a script with a main pipeline to load and classify our cohort was also included as extra features. This script was implemented to be used from command line and can handle several hospital files to be studied individually and as a unique cohort. Outputs of the package, if used as script pipeline, were CSV files with patients’ main data and their classifications and, optionally, an HTML report with several tables, graphs and interactive elements.

The HTML report generated by Andiacare constitutes an interactive data visualization and analysis environment designed to support clinical decision-making. This comprehensive tool incorporates multiple advanced features to facilitate real-time data exploration and interpretation. The platform includes dynamic filtering capabilities that enable users to select specific patient subcohorts based on various parameters, including clinical characteristics, time periods, and glycemic control levels. These filters allow for rapid customization of the dataset being analyzed to address specific clinical questions or research objectives. Interactive visualization tools form a core component of the system, featuring time-series plots, scatter diagrams, and histograms. These graphical representations support detailed exploration of both individual patient trajectories and group trends. A notable feature is the hover-to-detail functionality, which provides instant access to specific data points when navigating through the visualizations. The report incorporates comparative dashboards that simultaneously display patient stratification results according to different algorithms. This functionality enables clinicians to compare and contrasts classification systems, with the added capability to reorganize views based on clinical priorities. The system also generates dynamic tables containing all key glycemic metrics (TIR, TAR, TBR, CV, GMI, and scan frequency), which can be sorted according to various parameters and exported for further analysis or reporting purposes. Automated descriptive modules generate real-time summaries of the most relevant findings, with outputs that automatically adapt to reflect the currently selected data parameters. This adaptive functionality ensures that clinicians always have access to pertinent information tailored to their specific area of focus. This integrated environment provides clinicians with a powerful tool for exploring patient data in real time, testing different stratification approaches, and conducting comprehensive analyses—all without requiring external data processing. The system’s design emphasizes both clinical utility and workflow efficiency, supporting evidence-based decision-making at the point of care.

The Andiacare platform has been designed as a wrapper manager for different device sources. Currently, the majority of the pediatric population in Andalusia uses the Abbott Freestyle device due to public health system strategies, but the system is designed to accommodate other formats and could be expanded to include other devices.

### 2.6. Statistical Analysis

From the wide range of managed fields, only eight raw fields are statistically analyzed; the remining are used in charts or included in tables for interpretation purposes. These eight studied fields are (1) TIR; (2–3) TAR (1,2); (4–5) TBR (1,2); (6) Mean daily scan reads; (7) Age and (8) source center size. For all of them, some basic statistics are programmatically calculated, such as mean, minimum, maximum, quartiles 1, 2 and 3, standard deviation (SD) and the count (N) of available entries and missing values. Third parties’ criteria are also calculated like hospital sizes and age, which were analyzed using thresholds specified by SWEET [3]. For age, three thresholds were established: (a) 4–6; (b) 6–12; (c) >12 years and for hospital sizes: (a) large centers (>150 patients); (b) medium centers (50–150 patients), and (c) small centers (<50 patients). Basic statistics are also calculated for sub-cohorts obtained by applying age criteria. Other third parties’ criteria implemented was the ATTD targets for TIR, TAR (1 and 2) and TBR (1 and 2) which is used to identify sub-cohorts and is offered into tables with achieves and no achieves counts and percentage. A correlation study between the named fields is calculated for the full cohort and all sub-cohorts obtained by applying ATTD partial, or absolute, targets achievement.

Data analysis was conducted utilizing R version 4.4.2 (R Core Team, 2020), an open-source statistical software package (available at https://www.r-project.org, accessed on 14 May 2022). Normality of the study variables was assessed using the Shapiro–Wilk test. Continuous data are expressed as mean ± standard deviation (SD) for normally distributed variables or as median (interquartile range [IQR]) for non-normally distributed variables. Comparative analyses were performed using the *t*-test for normally distributed data and the Wilcoxon signed-rank test for non-normally distributed data. Correlation analyses were conducted using Pearson’s correlation coefficient for parametric variables and Spearman’s correlation coefficient for nonparametric variables. Categorical variables were evaluated using the chi-square test. A significance threshold of *p* < 0.05 was applied for all statistical tests. To account for multiple comparisons, *p*-values were adjusted using the Benjamini–Hochberg correction.

## 3. Results

### 3.1. Description of the Glucometric Data

**Figure 1:**
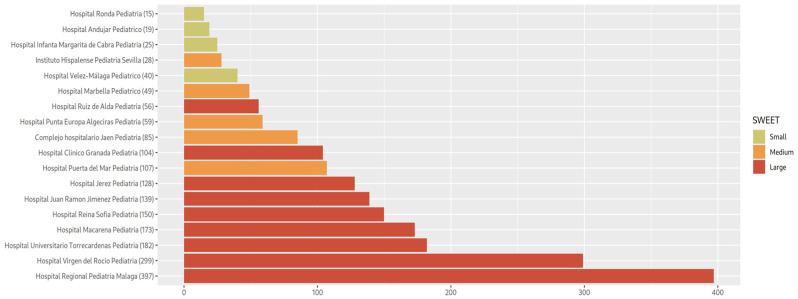
Number of patients per health center, colored by their center size scale by SWEET criteria.

The population-based study was drawn from 18 pediatric hospitals located in the Southern region of Spain during 1 November to 30 November 2022. In total, 2215 patients (1315 patients with ages between 12 and 18 years; 776 patients between 6 and 12 years of age and 124 patients between 4 and 6 years of age). With regard to hospital size, 50% of the patients were followed in large hospitals, 27.78% in medium-sized hospitals, and 22.22% in small-sized hospitals (Figure 1).

### 3.2. Glucometric Control Using Andiacare Algorithm

**Figure 2:**
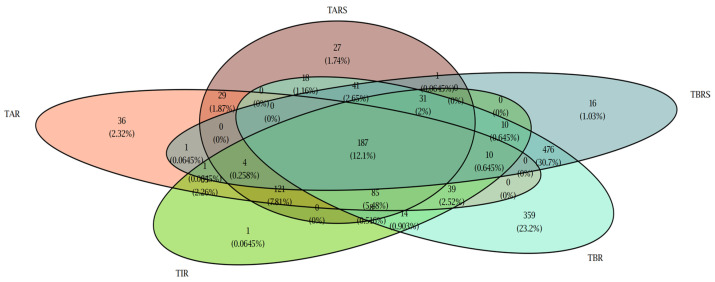
Graphical representation of the number and percentage of patients achieving control target for each glucometric variable. The colors indicate TIR (green), time above range (TAR/TARS, red tones), and time below range (TBR/TBRS, blue tones).

The results showed that 25.8% (572/2218) of the cohort achieved the recommended TIR; only 4.0% (89/2218) achieved glucose controls in target levels (below 154 mg/dL) and SD below 29 mg/dL, while 43.4% (960/2218) obtained recommended CV below 36%. Dividing TBR in level 1 and 2, 60.6% (1341/2218) of the patients achieved the recommended %TBR1, and 39.53% (821/2077) achieved the recommended %TBR2. Likewise, 26.1% (578/2218) of the patients achieved the recommended %TAR1 and 28.07% (583/2077) achieved the recommended %TAR2). In total, 9.5% (211/2218) of the patients achieved all target parameters of glucometric control; however, when level 1 targets (TIR, TBR1 and TAR1) were considered, this percentage increased to 18.9% (Figure 2).

The average of each variable and the percentage of patients who achieved the goal in the different age groups is shown in Table 1 and Table 2.

**Figure 3:**
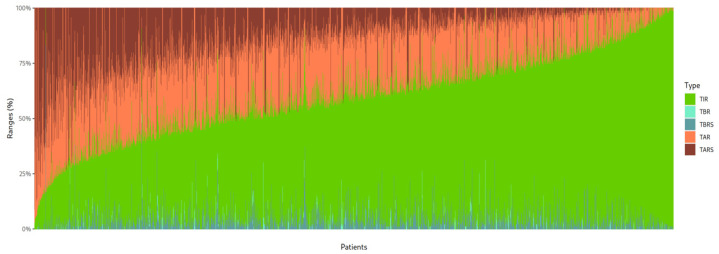
Stacked bar plot representing the distribution of glycemic ranges for each patient, ordered from lowest to highest time in range (TIR). The colors indicate TIR (green), time above range (TAR/TARS, red tones), and time below range (TBR/TBRS, blue tones).

A notable aspect in the graphic representation is how the space not occupied by TIR is occupied by TAR and not by TBR (Figure 3).

### 3.3. Correlation Between Glucometric Parameters and Number of Daily Scans

In all patients, CV was positively correlated with Glucose Management Indicator (GMI) (R = 0.6, *p* = 0.005). In patients who met all targets, the mean number of daily scans showed positive correlation with GMI (R = 0.34, *p* = 0.005), TAR1 (R = 0.33, *p* = 0.005) and TAR2 (R = 0.27, *p* = 0.005), but the mean number of daily scans showed negative correlation with TIR (R = −0.34, *p* = 0.005) and the age of the patients (R = −0.17, *p* < 0.05). On the other hand, in patients who did not meet targets, mean daily scans showed a negative correlation with GMI (R = −0.20, *p* = 0.005) and all control parameters, except for TIR, which showed a positive correlation (R = 0.23, *p* = 0.005). In patients who did not meet targets a negative correlation between the age of the patient, TIR (R = −0.17, *p* = 0.005) and mean daily scans (R = −0.32, *p* = 0.005) was reported, while age showed a positive correlation with other control parameters such as TIR, TAR1, TAR2, TBR1, TBR2, and GMI (*p* = 0.005), respectively.

### 3.4. Comparison Between Andiacare Platform and GRI Algorithms

**Figure 4:**
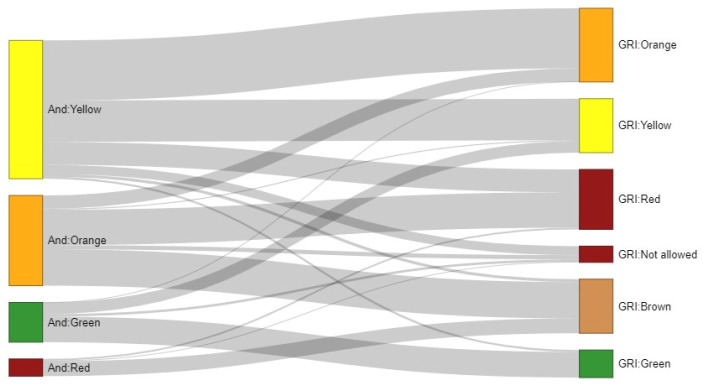
Sankey diagram illustrating the correspondence between patient classifications obtained with the Andiacare (left) and GRI (right) classifiers. Flows represent the number of patients sharing a given pair of tags, with node colors corresponding to the categories of each system. Some patients lacked data for certain parameters required by GRI and were labeled as “Not allowed” for this classifier, but they could still be classified using Andiacare—hence their inclusion in the diagram.

The percentage of patients achieving the green category in Andiacare is 20%, compared to 10% for the GRI algorithm; the cohort’s classification according to the Andiacare algorithm is displayed along with a comparison to the GRI-previously described classification (Figure 4).

## 4. Discussion

To the best of our knowledge, this is the first study that analyzes glucometric control in a very large pediatric patient population with T1D. Until now, the status of pediatric children with T1D had been analyzed in smaller samples and not distributed by age and hospital size [2,5,10,16,17,18,19,20]. The present study, which analyses the glucometric control of children with T1D in Andalusia, shows for the first time the percentage of pediatric patients in treatment with MDI and isCGM who achieve goals proposed in the 2019 consensus with regard to TIR, TAR1, TAR2, TBR1, and TBR2, in a cohort of patients with T1D in the Southern Region of Spain.

For such analysis, an interoperable platform, Andiacare, has been designed; this innovative tool allows sample categorization and facilitates the management of large patient samples and the identification of those who require targeted intensification.

Only a small proportion of pediatric patients with T1D achieved the recommended international targets for TIR. Goals related to TBR1 appeared to be relatively attainable, whereas those for TBR2 remained more difficult to accomplish. In contrast, hyperglycemia, reflected in both TAR1 and TAR2, emerged as the predominant challenge, limiting the ability of patients to maintain adequate TIR. Taken together, these findings suggest that hyperglycemia is the major clinical issue in this population, highlighting the need for further strategies to improve glycemic management.

In our cohort, the average TIR was 57.73%. Regarding the glucometric data published by other series, the results of Cherubini et al. showed a mean of 40% of TIR in the group with is CGM. The same group established that patients treated by MDI and isCGM showed significantly higher median values of TAR (44%) [10,21]. While the Italian group showed a percentage of participants with MDI and isCGM achieving TIR > 70% of 8.3%, our study showed a higher percentage of children who reached this target of TIR (25.79%) [10]. In another study conducted by Urakami et al., the mean percentage of TIR and TBR in children and adolescents with T1D using isCGM were 50.7 ± 12.2% (23–75%), and 11.8 ± 5.8% (2–27%), respectively [5].

In another report, Edge et al. showed that the mean of TIR in pediatric patients aged 4–17 years with type 1 and type 2 DM was 50%, and Campbell et al. showed that the mean of TIR was 46% in pediatric T1D patients aged 4–17 years. A recent report of an 11-center cross-sectional study in a large group of children with T1D using CGM with non-automated insulin delivery systems demonstrated that the median frequencies of TIR were 49%, 56%, 56%, and 61% in patients on isCGM with MDI, on rtCGM with MDI, on isCGM with an insulin pump, and on rtCGM with an insulin pump, respectively [18,22]. In these studies, there was a wide interindividual variation in the frequencies of TIR, while in our cohort a higher TIR value was achieved in all age groups. Nevertheless, our study, as well as all published studies, reflected lower than the recommended TIR goal values. It can be concluded that it is challenging to achieve the recommended TIR goal of >70% in pediatric patients treated with MDI and isCGM.

Children and adolescents with T1D are likely to have high magnitudes in postprandial glucose levels and remarkable interindividual and day-to-day glycemic variations. Therefore, it may be substantially difficult to maintain the glucose level of 70–180 mg/dL to more than 70% in the management of pediatric patients with type 1 diabetes. The recommended TIR target proposed by the ATTD panel is more frequently achieved with an automated insulin delivery system [5,9]. Based on the glucose metrics reported for the four therapeutic strategies, few children with T1D were able to reach a TIR > 70%, while the number increases substantially if the target is considered at TIR > 60%. As a result, the authors questioned the recommended %TIR. We still do not have studies correlating medium to long-term complications with glucometric variables. Therefore, long-term monitoring of currently monitored cohorts becomes necessary, although international consensus recommendations maintain a TIR goal of over 70% for patients under 25 years old [9,10]. Similarly to other published studies, TIR was highly inversely correlated with TAR and HbA1c level but not with TBR [9,10].

This platform not only allows for the categorization of each glucometric variable individually but also enables the integration of glucometric variables, generating algorithms (Andiacare and GRI) (Figure 4). Neither algorithms can be directly correlated because GRI is based on a numerical function (linear combination) of hyper- and hypoglycaemia weighted times. On the other hand, Andiacare is based on achievement, or not, of time in ranges objectives. Since the classification strategies have essential discrepancies, this leads us to compare only the states that aggregate the results of each classifier. As both algorithms are based on expert consensus rather than the evolution and clinical evidence of the patients, the only way to confirm the accuracy of each would be to compare a long-term temporal evolution of the same cohort using both algorithms.

With regard to hospital size, the current analysis shows that the gross distribution of patients is equal between large hospitals with respect to medium and small hospitals combined. This analysis shows a more homogeneous distribution in our area with respect to the worldwide distribution previously reported, in which a bulk of patients are followed in medium-sized hospitals (45.6%), 30.2% in large-sized hospitals, which is a higher number than in our sample, and 23.2% in small-sized hospitals [4]. This distribution is the result of our health organization’s efforts to address the diabetes epidemic on a global scale by means of a public health system. Because of the high percentage of rural residents and population dispersion in our area, healthcare is provided at the healthcare facility closest to the patient to ensure accessibility. No differences have been found in glucometric results in relation to the size of the center that cares for patients.

Regarding adherence to the use of the sensor, the results show that the group between 12 and 18 years of age had a lower adherence to the sensor and presented a significantly lower TIR, a higher TAR1 and 2 and a higher TBR1 and 2. In fact, this group had the lowest number of patients who achieved the targeted goals for these four parameters.

Regarding the adhesion of the sensor, some studies showed that the mean scanning frequency was approximately 15 times/day in T1D. A European analysis in a large population for T1D found a high scanning frequency of 16 scans/day in over 60 million glucose tests [5]. The mean scanning frequency was 11.5 times/day in Japanese children and adolescents with type 1 diabetes with isCGM [5,23]. In the present study, the average number of scans was 14.57/day, decreasing as the age of the patient increased. Several reports have demonstrated that a high frequency of glucose scanning in isCGM contributes to improvement of glucometric control and TIR [5,24], with a reduction in glycemic variability, HbA1c, TAR, and TBR [5,19,20,25]. The same trends, with unique nuances, are observed in both Spanish and global data [20,25]. However, our study showed that in patients who met all targets, the number of scans was directly correlated with increased TAR2 and GMI, whereas in those with poorer control, a positive correlation was observed with TIR. This could be explained by a ceiling effect in the number of scans: in poorly controlled patients, performing scans appeared to be inherently beneficial, while in well-controlled patients, the most efficient approach seemed to involve achieving the best possible TIR with the minimum number of scans required. This conclusion is consistent with the results of Leiva-Gea et al., which showed that HbA1c was higher at lower scan rates and decreased as the scan rate increased to 15–20 scans, after which it rose at higher scan rates [25,26]. An interesting point to discuss is the similarity as a group in terms of adherence measured by the number of daily scans. In the group of individuals who meet targets, there is a negative correlation between the number of scans and TIR, unlike the group that does not achieve glucometric targets, in which the number of scans has a positive correlation with TIR. This information is crucial for a better understanding of the intervention and education needs in diabetes, especially in optimizing the frequency of monitoring with intermittent glucose sensors.

In another study conducted by Urakami et al., the mean percentage of TIR and TBR in children and adolescents with T1D using isCGM were 50.7 ± 12.2% and 11.8 ± 5.8%, respectively. Notably, only 27.3% of patients achieved the recommended CV ≤ 36%, and these individuals exhibited higher TIR and lower TBR compared with patients with CV > 36% [27]. CV is considered a complementary metric for interpreting CGM data in pediatric T1D. When compared with the cohort of Urakami et al., a considerably larger proportion of patients in our study (43.4%) achieved CV ≤ 36%.

There was no significant correlation between the scanning frequency and patient’s age [28]. Related to age, children < 12 years of age showed the best metabolic control and the most frequent use of the device, metabolic control is deteriorated with age, and the greater number of device scans was in correlation with better metabolic control in all age groups [28]. At this point, our study reflected poorer adherence to treatment and fewer scan reads in older patients, which could explain the very limited glucose controlled achieved in prior studies and in the present study where patients > 12 years of age achieved higher TAR and TBR values and lower TIR values.

The Andiacare patient categorization platform shows, in less than 7 s, the glucometric results of patients distributed by hospitals and age groups, which allows us to geographically evaluate and allows the design of strategies by age groups and hospitals adjusted for size.

Until now, the data used for analysis were found in passive patient management platforms such as the LibreView platform, which shows the results of the patients individually and after active entry in the patient profile. Such passive platforms (LibreView) do not allow group and large-scale analyses that enable us to evaluate and design management policies. Until now, the management of patient appointments has been unrelated to the patient’s glucometric assessment.

This type of tool could potentially support changes in care delivery by enabling real-time stratification, regardless of the other unrelated physician’s appointments. Good data linkage and timely data availability to frontline health staff have the potential to transform care delivery [17]. The utility of such tools, applied on a large scale to evaluate patients in real time, remains valid at the present moment, when we are experiencing an effervescence of different devices that require rapid evaluation. These tools that allow clinicians and managers to assess devices, health programs, virtual care, appointment frequency, and the duration of face-to-face and virtual consultations in order to link expenses with the prioritization of therapeutic scalability for patients who need it most. It also aims to identify the ceiling effect in the glucometric results of certain actions and therapies.

This paper gains vital importance at the current moment, when multiple guidelines position closed-loop therapy as the first indication in pediatric patients with T1D, necessitating tools for scalability planning and outcome evaluation [29,30,31].

Several limitations should be considered when interpreting these results. The analysis is based exclusively on data extracted from the LibreView platform, which may introduce information bias due to its predefined data structure and limited variable flexibility. This limitation underscores the need to utilize raw data from all devices, enabling comprehensive data exploitation and comparability. The retrospective nature of the study design may introduce potential selection biases and unmeasured confounding factors. Variations in sensor usage duration among participants could potentially influence glycemic metrics, although we implemented standardized analysis windows to minimize this effect. The database we used is extensive and anonymized; only age and hospital size were available, which prevented cross-referencing with other clinically relevant variables (such as exact disease duration and gender) or psychosocial factors that might have influenced the outcomes. Another limitation is that multivariate analyses could not be performed, not only due to the low number of variables but mainly because of the structural restrictions of the anonymized database. Finally, as our cohort consisted exclusively of patients using MDI and isCGM, caution is warranted when attempting to extrapolate these findings to other treatment modalities such as insulin pumps or automated insulin delivery systems.

Future research should address these limitations through prospective study designs that incorporate data from multiple platforms and implement standardized protocols for sensor use. Such approaches would help validate our findings and enhance their applicability across different clinical settings and patient populations. Despite these limitations, our study provides valuable insights into glycemic control patterns among pediatric T1D patients in Southern Spain and identifies important areas for improvement in diabetes management strategies.

## 5. Conclusions

To the best of our knowledge, this study constitutes the largest analysis of pediatric T1D patients from Southern Spain. The findings indicate that only a limited proportion of pediatric T1D patients achieved the recommended TIR and TAR targets under MDI and isCGM therapies. The results suggest a notable association between TIR and TAR levels, with age also emerging as a relevant factor in the attainment of glycemic targets. These observations support the continued investigation into novel algorithms that incorporate both glucometric and non-glucometric parameters to refine patient categorization and facilitate more tailored therapeutic strategies, which may contribute to improved outcomes within the constraints of healthcare sustainability.

The interpretation of these results should consider the specific treatment modality of the cohort, which was restricted to MDI and isCGM. Consequently, extrapolation to other therapeutic approaches, such as insulin pumps or automated insulin delivery systems, may not be warranted.

## Figures and Tables

**Table 1:** Age-group distribution of glucometric variables and daily scan and percentage of patients achieving the proposed ATTD target for each glucometric variable.

Age Group (Size)	Mean ± SD						Meet ATTD Targets (Size)				
	TAR2	TAR1	TIR	TBR1	TBR2	Daily Scan Reads	TAR2	TAR1	TIR	TBR1	TBR2
Age 12–18 (1315)	17.27 ± 16.31	40.43 ± 20.35	54.2 ± 19.48	4.65 ± 5.83	4.34 ± 5.58	9.12 ± 8.04	23.57% (310)	23.04% (303)	21.29% (280)	55.36% (728)	34.29% (451)
Age 6–12 (776)	12.14 ± 11.68	34.49 ± 18.21	62.07 ± 17.88	3.45 ± 4.29	3.23 ± 4.05	13.75 ± 11.76	30.67% (238)	31.06% (241)	32.99% (256)	68.43% (531)	40.72% (316)
Age 4–6 (124)	14.04 ± 13.59	35.82 ± 18.76	60.46 ± 17.39	3.72 ± 4.78	3.63 ± 4.72	20.38 ± 18.13	28.23% (35)	27.42% (34)	29.03% (36)	66.94% (83)	43.55% (54)
TOTAL (2215)	15.29 ± 14.89	38.09 ± 19.74	57.73 ± 19.12	4.18 ± 5.31	3.91 ± 4.07	11.42 ± 10.76	26.32% (583)	26.09% (578)	25.82% (572)	60.59% (1342)	37.07% (821)

Paired *t* test. The data are presented as mean ± SD.

**Table 2:** Comparison of glucometric variables and mean readings according to age group. Group 1 (4–6 years), Group 2 (6–12 years), and Group 3 (12–18 years).

	Age Group			*p* Value		
	Group 1	Group 2	Group 3	Group 1–2	Group 1–3	Group 2–3
TAR2 (%)	14.04 ± 13.59	12.14 ± 11.68	17.27 ± 16.31	0.279	0.056	2.58 × 10^−7^
TAR1 (%)	35.82 ± 18.76	34.49 ± 18.21	40.43 ± 20.35	0.637	0.036	8.06 × 10^−7^
TIR (%)	60.46 ± 17.39	62.07 ± 17.88	54.2 ± 19.48	0.508	0.004	1.56 × 10^−9^
TBR1 (%)	3.72 ± 4.78	3.45 ± 4.29	4.65 ± 5.83	0.859	0.859	2.66 × 10^−4^
TBR2 (%)	3.63 ± 4.72	3.23 ± 4.05	4.34 ± 5.58	0.703	0.278	5.96 × 10^−4^
Mean Reads	20.38 ± 18.13	13.75 ± 11.76	9.12 ± 8.04	0.004	5.43 × 10^−6^	1.05 × 10^−12^

Paired *t* test. The data are presented as mean ± SD.

## Data Availability

Full data has been anonymized and is openly available for using in GitHub repository (https://github.com/fmjabato/andiacarepackage, accessed on 23 September 2022).

## Prior Presentation

Parts of this study were presented in the oral presentation session of the 34th National Congress of the Spanish Society of Diabetes, Valencia, Spain (2023), and 45th National Congress of the Spanish Society of Pediatric Endocrinology, Palma de Mallorca, Spain (2023).

## Abbreviations

The following abbreviations are used in this manuscript:

T1D	Type 1 diabetes
T2D	Type 2 diabetes
ATTD	Advanced Technologies and Treatments for Diabetes
TIR	Time In Range
TAR1	Time Above Range level 1
TAR2	Time Above Range level 2
TBR1	Time Below Range level 1
TBR2	Time Below Range level 2
CV	Coefficient of variation
MDI	Multiple daily injections of insulin
CSII	Continuous subcutaneous insulin infusion
CGM	Continuous glucose monitoring
rtCGM	Real-time continuous glucose monitoring
isCGM	Intermittently scanned continuous glucose monitoring
GRI	Glycemic Risk Index
GMI	Glucose Management Indicator
SD	Standard deviation
IQR	Interquartile range
